# Assessing the Effects of Basic Medical Science Courses on the Knowledge and Attitude towards Antibiotic Usage among Pre-Professional Students in Saudi Arabia

**DOI:** 10.3390/pharmacy9020108

**Published:** 2021-05-30

**Authors:** Ismail Memon, Azzam Abdulaziz Alrashid, Hamad Saadi Alshammari, Dur-e-Shewar Rehman, Zeeshan Feroz, Abdulaziz Nagro, Rakan Alotaibi, Muath Alsalmi, Muhammad Anwar Khan, Abdulmohsen Alkushi, Syed Faisal Zaidi

**Affiliations:** 1 Basic Science Department, College of Science and Health Professions, King Saud bin Abdulaziz University for Health Sciences, Riyadh 11481, Saudi Arabia; rehmand@ksau-hs.edu.sa (D.S.R.); ferozz@ksau-hs.edu.sa (Z.F.); kushia@ksau-hs.edu.sa (A.A.); 2 King Abdullah International Medical Research Center, Riyadh 11481, Saudi Arabia; 3 College of Medicine, King Saud bin Abdulaziz University for Health Sciences, Riyadh 11481, Saudi Arabia; alrashid497@ksau-hs.edu.sa (A.A.A.); alshammari396@ksau-hs.edu.sa (H.S.A.); 4 College of Medicine, King Saud bin Abdulaziz University for Health Sciences, Jeddah 21423, Saudi Arabia; nagro024@ksau-hs.edu.sa (A.N.); alotaibim007@ksau-hs.edu.sa (R.A.); alsalmi009@ksau-hs.edu.sa (M.A.); khana@ksau-hs.edu.sa (M.A.K.); 5 King Abdullah International Medical Research Center, Jeddah 21423, Saudi Arabia; 6 Department of Pharmacology, School of Medicine, Batterjee Medical College for Sciences and Technology, Jeddah 21442, Saudi Arabia

**Keywords:** antibiotics, health science students, knowledge, attitude, Saudi Arabia

## Abstract

The curriculum of medical schools includes courses on antibiotics. Therefore, it is worth exploring information related to the knowledge and attitudes about antibiotics. In this cross-sectional study the questionnaire was administered to the undergraduates in two phases, before and after attending the basic medical science courses. The data were collected on demographic variables, source of antibiotics, level of knowledge, and changes in attitude statements. Data analysis was implemented using SPSS. The mean age of participants was 19.87 and 20.15 in phases I and II, respectively. Most of the participants’ parents had education at the university level and a monthly income above 15,000 SAR. Generally, students had good knowledge and attitude about antibiotics. A significant improvement in students’ knowledge in phase-II was noticed in “level of knowledge” (*p*-value = 0.044), “paracetamol is considered an antibiotic” (*p*-value < 0.001) and “overuse of antibiotics can cause antibiotics resistance” (*p*-value = 0.003). The overall knowledge and attitude of pre-professional students were good in both phases, but their attitude did not improve at a significant level in phase-II. There is a need to put more focus on antimicrobial therapy in their training.

## 1. Introduction

Antibiotics are used to treat bacterial infections. Irrational use of antibiotics results in antibiotic resistance. Internationally there are concerns that the world is losing its powerful arsenals against bacteria due to resistance [1]. Obtaining antibiotics without prescription is more common in developing countries and is becoming a problem in developed countries, too [2]. In Saudi Arabia, there is an irrational use as a result of lack of antibiotics knowledge; and the public frequently contacts medical students and allied health workers for the use of antibiotics [3,4,5]. Therefore, the training of health science students and health care workers should focus on improving their knowledge, attitude, and practices about antibiotics. Such endeavors will encourage the cautious use of antibiotics [6,7]. Previous studies indicated that the medical curriculum that focused on antibiotics’ rational use significantly improved students’ knowledge of antibiotics and discouraged inappropriate use of antibiotics in their clinical practice [8,9].

The College of Science and Health Professions (COSHP) at King Saud bin Abdulaziz University for Health Sciences (KSAU-HS) offers a two-year pre-professional program (PPP) consisting of four semesters (Table 1). In the last semester of PPP, basic medical science courses, including pharmacology, are completed [10]. The pharmacology and microbiology courses included the classification, spectrum and specificity, mechanism of action, adverse effects, indications and contraindications about antibiotics. This study aimed to compare the level of knowledge and attitude about antibiotics use among pre-professional students (PPS) before (phase-I) and after (phase-II) completion of basic medical science courses. Thus, the data would serve as a baseline to see the basic medical science courses’ effects on antibiotics’ knowledge and attitude among PPS. 

## 2. Materials and Methods

### 2.1. Study Design and Sample

This is a prospective survey in which an approved validated self-administered questionnaire was used [11]. The Institutional Review Board (King Abdullah International Medical Research Center, Riyadh, Saudi Arabia) approved the study (Study No: RC19/021/R). This study was conducted at the COSHP, male and female colleges, Riyadh campus during the academic year 2018–2019. The questionnaire was administered to the students in two phases. In phase-I, the students were invited to fill the questionnaire before the beginning of the fourth semester, in which basic medical science courses, including pharmacology, were offered. In phase-II, the questionnaire was administered to the same students upon completing the fourth semester. 

The number of participants was about 800 students in phase-I and about 700 students in phase-II. This number was calculated to get an adequate sample size for this study, representing the student population. The response distribution was assumed to be 50%, and the confidence interval was established at 95% with a 5% margin of error. The sample size was computed using the Raosoft software to be 260 in phase-I and 249 in phase-II, accounting for a 15% non-response rate using the non-probability convenience sampling technique [12]. The sample size was increased to make it more representative of the population and decrease the risk of selection bias. Eventually, 857 students were included in this study to reduce selection bias and achieve the target population’s appropriate representation. In phase-I 433 and phase-II, 423 students were surveyed. In phase-I, second-year PPP students, before taking the basic medical science course and ready to participate in the study, were included. For phase-II, the students who attended pharmacology and basic medical science courses in the PPP and were willing to participate in the survey were included. The students who did not attend the basic medical courses including, pharmacology and microbiology, were excluded in the phase-II survey.

### 2.2. Development of the Questionnaire

The questionnaire previously used by Ling et al. was improved to suit this study’s population [11]. In the present study, the questionnaire was administered twice (phase-I and phase-II) to the same population and was modified accordingly to match the needs of the study. The questionnaire contained four parts: Part 1 focused on the students’ demographic characteristics and their parents’ educational and financial backgrounds. Part 2 inquired about antibiotic usage over the past six months and its source. Part 3 related to the knowledge of antibiotics, and it consisted of 14 statements. This part assessed the subjects’ change in knowledge regarding the role, identification, and dangers of antibiotics and the completion of the treatment course. Participants were asked to select one of three options provided: “Yes”, “No”, or “Not Sure”. Each correct response provided one score, while incorrect and not sure answers yielded no score. The highest achievable score was 14. Part 4 consisted of nine statements, and it was designed to evaluate students’ attitudes about antibiotics. The Likert-type scale was used to measure students’ attitudes. Positive responses/attitudes would denote the suitability of using antibiotics, whereas negative responses/attitudes would imply the inappropriateness of such use. For the attitude part, the unsure option was considered as a wrong attitude, and the level of attitude was calculated based on the Malaysian study [11]. The options “Disagree” for statements 1 to 6 and 9 and “Agree” for statements 7 and 8 indicated a positive response/attitude. Content and Arabic translation face validations were achieved [3]. Reliability testing regarding attitude was executed in the previous pilot study with the Cronbach’s α value of 0.76.

### 2.3. Data Analysis

The scoring system used to assess the knowledge of the students was depending on their responses. The total score of knowledge was classified into three categories: good (10–14), moderate (5–9), and poor (0–4). Data analysis was accomplished using IBM Statistic SPSS (SPSS Inc., Chicago, IL, USA) version 25.0. Descriptive analysis was conducted to calculate the study results, such as demographic characteristics, knowledge and attitudes toward antibiotic usage, and recent use of antibiotics. The effect of demographic characteristics on attitude and knowledge was checked by applying Fisher’s exact or Chi-square tests wherever suitable, and *p*-values were obtained for each test. The level of statistical significance was set at *p*-value < 0.05. The demographic characteristics of phase I were used for calculating the *p*-values and assumed that both phases are the same population. 

## 3. Results

This study’s target population filled the questionnaires at the beginning of semester-4, phase-I, and after completing the basic medical science courses, phase-II. As depicted in Table 1 for phase-I and phase-II, the mean ages of male and female participants were 19.87 ± 1.60 and 20.15 ± 1.85, respectively. Amongst the 433 students who participated in phase-I, 53.8% were males, and 46.2% were females. Comparably in phase-II, 423 students took part in the survey, where 55.3% were males, and 44.7% were females. Most of the participants’ parents’ highest educational status was College/University level (phase-I, 67% fathers and 60.9% mothers) and (phase-II, 73.8% fathers and 62.1% mothers). Furthermore, most parents (67.9% in phase-I and 72.8% in phase-II) had a monthly income above 15,000 SAR. Table 2 shows the usage and source of antibiotics where176 (40.6%) students gave a history of recent antibiotics use within the last six months for phase-I and 147 (34.8%) for phase-II. Of those students, 78.8% (phase-I) and 80.7% (phase-II) stated that the source of obtaining the antibiotics was via prescription; and there was no significant difference between the two phases when the McNemar-Bowker test was applied.

Table 3 compares the level of knowledge of antibiotics between phases I and II. A change in knowledge level came out to be significant (*p*-value = 0.044) when the McNemar-Bowker test was applied, indicating an improvement in the level of knowledge from phase-I to phase-II. McNemar-Bowker test was used in Table 4 to study the association between demographic characteristics and change in knowledge level. None of the demographic characteristics showed a significant association with the level of knowledge. Nevertheless, female students showed some improvement (phase-I 39.8%, phase-II 57.3%) in the level of knowledge in comparison to males (phase-I 41.8%, phase-II 43.8%). Associations between the other demographic characteristics such as the father’s educational status, mothers’ educational status, and parents’ monthly income were insignificant and showed no noticeable difference.

Change in knowledge statements was analyzed using McNemar-Bowker test and is represented in Table 5. As for the knowledge of antibiotics’ role, around 91% of students in both phases correctly identified that antibiotics are used to kill bacteria. In addition, in both phase-I (69.5%) and phase-II (72.8%), students correctly identified that antibiotics are not used to treat viral infections. However, around 53% of the participants, in both phases, incorrectly stated that antibiotics are indicated to relieve pain/inflammation. Furthermore, the students gave a mixed response to the statement, “antibiotics are used to stop fever”, yet their correct response improved in phase-II (42.8% phase-I and 46.8% phase-II). Regarding penicillin, most students correctly identified that it is an antibiotic (76.3% phase-I, 80.5% phase-II). On the other hand, around 43.5% of students in phase-I were unsure if aspirin is a new generation of antibiotics compared to 31.6% of students in phase-II. Furthermore, regarding the question asking if paracetamol is considered an antibiotic, 42.4% gave a correct response, which significantly improved in phase-II (56.5%) (*p*-value < 0.001). As for the dangers of antibiotics questions, the only significant statement was regarding the antibiotic resistance phenomenon. In phase-I, 65.4% of students correctly identified that overuse of antibiotics can cause antibiotics resistance; their response significantly improved (*p*-vale 0.003) in phase-II (76%). For the statement “completion of the treatment course”, the students correctly stated that they would not stop taking a full course of antibiotics if their symptoms improve (74.9% phase-I, 76.0% phase-II). They also agreed that treatment effectiveness is reduced if the full course of antibiotics is not completed (82.4% phase-I and 84.2% phase-II).

Table 6 represents the change in attitude towards antibiotics between phases I and II. McNemar-Bowker test was applied to analyze the change. Most of the students, in both phases, were aware of the misconception of giving one’s antibiotics to a sick family member and recommending antibiotics to sick friends and family. However, their attitude regarding giving one’s antibiotics to a sick family member decreased in phase-II. Similarly, although most students were convinced to take antibiotics according to the instructions on the label, a drop in this conviction was observed in phase-II. Moreover, most of the study subjects had a positive attitude towards using antibiotics in common cold/flu, expecting antibiotics prescription from a doctor if suffering from flu, stopping antibiotic course on feeling better, checking the expiry date before use, keeping antibiotics stock at home to be used in an emergency, and using leftover antibiotics for respiratory illness. Nevertheless, their attitude did not improve at a significant level in phase-II. 

## 4. Discussion

Many studies around the world have been conducted on knowledge, attitude and usage of antibiotics. These studies targeted diverse populations, and the outcomes of the conducted studies were also different depending upon the variables and the areas of studies. This study evaluates the effects of basic medical sciences courses, including basic pharmacology, on PPSs’ knowledge and attitude before and after taking these courses. The present study is the first study of its kind in Saudi Arabia conducted on PPS.

Table 1 shows that the number of male participants is higher than the female participants, which coincides with their numbers in the male and female colleges. Our data show that about 70% of parents have a monthly income of ≥15,000 Saudi Arabia Riyal, which is almost similar to the study from Jeddah [3]. These findings are closely related to a financial report published in the Saudi Gazette in July 2020, which indicated that a Saudi family’s average monthly income is 14,823 SAR [13]. 

Globally, more than 50% of antibiotics are obtained without a prescription [14]. The worst scenario is where antibiotics are freely available without legal obligations [2]. In 2018, the Saudi government implemented a strict law against the selling of antibiotics without a prescription. In non-compliance with the law, the seller/pharmacy could face license suspension, imprisonment, and fines [15]. This study was conducted after implementing the antibiotics regulatory law; consequently, obtaining antibiotics without prescription showed a reduced trend from 21.2% in phase-I to 19.3% in phase-II. There was no significant difference between the two phases; however, most of the students in both phases, obtained antibiotics through a lawful source (Table 3).

The importance of knowledge about antibiotics among medical students and allied health workers has been identified [6,7]. Surveys from Jeddah and Riyadh showed poor to moderate knowledge about antibiotics [3,16]. Moreover, the medical curriculum that focuses on the appropriate use of antibiotics significantly improves students’ knowledge of antibiotics [8,9]. In the same context, this study shows a significant improvement in the students’ knowledge (level of good knowledge) after attending the basic medical science courses in phase-II (Table 3).

Table 4 shows that none of the demographic characteristics are significantly associated with the level of knowledge. Nevertheless, similar to a Caribbean study, females in this study showed a slightly better knowledge level [17]. We observed that the student’s parents’ higher education and income status are not significantly associated with their knowledge of the antibiotics. 

In this survey, the role of antibiotics against bacterial infections, viral infections, any type of infection, pain, and fever was analyzed (Table 5). Many studies have indicated confusion among students regarding the role of antibiotics [16,18,19]. Like the findings of a survey from Italy, more than 90% of students in this study, in both phases, correctly identified that antibiotics are useful against bacterial infections [20]. In addition to the flu, many other infections could be of viral origin, which does not need an antibiotic prescription. Regarding antibiotics’ role against viral infections, the majority of the students, in both phases, correctly identified that antibiotics are not used to treat viral infections. However, there are different responses to the antiviral activity of the antibiotics [3,9,20,21]. Therefore, the healthcare-related students, approached by common people for their ailments, must know that unnecessary antibiotic usage for viral infections creates side effects on the users’ health.

Furthermore, there are misconceptions and confusions among the students about using antibiotics as a painkiller [22,23]. Likewise, in the present study, more than 52% of students in both phases incorrectly stated that antibiotics are indicated to relieve pain/inflammation. Our students gave a mixed response to the statement that antibiotics are used to stop the fever, yet their correct response improved in phase-II. Such misconceptions were also observed in other studies [3,9,18,22]. Easy access to antibiotics and misconception regarding identifying penicillin as an antibiotic is common in Saudi Arabia [2]. In contrast to studies from Jeddah and UAE, and consistent with a study from Italy, most of the study participants identified that penicillin is an antibiotic [20]. Regarding identifying aspirin as a new generation of antibiotics, the same as described by Zaidi et al., 43.5% of our students in phase-I and 31.6% of students in phase-II were unsure [3]. The gaps in knowledge about antibiotics among medical students draw attention to emphasizing such issues in their courses. In this survey, only about half of the participants could correctly identify that paracetamol is not an antibiotic. This is the only question in “change in knowledge statements”, where students’ correct responses significantly improved in phase-II (*p*-value < 0.001) (Table 5). The possible reason for the wrong perception about paracetamol could be attributed to the fact that medications are sold with brand names, different from generic names, in Saudi Arabia. 

Another critical issue is antibiotics resistance due to over-usage. The surveys from different countries, including Saudi Arabia, showed most students knew that easy access and overuse of antibiotics cause antibiotic resistance [2,3,20,24,25]. To combat increasing antibiotic resistance trends, the Saudi Ministry of Health took effective measures [26,27]. In the present study, students’ correct response to the statement, “antibiotics’ overuse can cause antibiotic resistance” significantly increased (*p*-value = 0.003) in phase-II. It is expected that restrictions against over-the-counter selling of antibiotics and public awareness would reduce the antibiotic resistance problem in Saudi Arabia. It is common to observe that patients usually discontinue the antibiotics course when they feel better [3,18,22,28,29]. Nonetheless, Riyadh PPS had better knowledge about the completion of the antibiotic course. This could be attributed to the notion that big cities’ population dwellings are more aware of such issues [30,31].

A higher level of knowledge about antibiotics is usually related to a good attitude [9,16,32]. Simultaneously, published literature has highlighted discrepancies between knowledge levels and appropriate attitudes towards antibiotics [17,20]. The sharing of antibiotics has been identified as a global issue [33]. Table 6 represents the change in attitude towards antibiotics in phases I and II. Most of the students in both phases were aware of the misconception of giving one’s antibiotics to a sick family member; however, their attitude decreased in phase-II (*p*-value = 0.015). These findings match a study conducted in Singapore, where only 6.8% of respondents shared antibiotics with family members [34]. Safe and appropriate use of antibiotics must follow the instructions from the prescribing doctors, pharmacy personnel, or written on leaflets/labels. In this survey, most students, in both phases, agreed to take antibiotics according to the label; a drop in this conviction seemed in phase-II. This shows that Riyadh’s PPS students had a more positive attitude towards following the instructions for antibiotic use than those from Nepal and Pakistan [22,25]. The general public and health care workers are not clear about using antibiotics as a treatment for flu. The respondents worldwide had a diverse attitude towards using antibiotics in case suffering from the flu [3,11,18,29,35,36]. Like other studies, our students showed a positive attitude, in both phases, towards using antibiotics in flu [9,17,20,21]. The students’ positive attitude towards antibiotics in this study could be because Riyadh is the capital and biggest city of Saudi Arabia; social, economic, educational status, and general awareness among the population could be higher. It is also commonly observed that patients demand or expect antibiotics prescriptions from doctors when they have flu. To comply with patients’ satisfaction, social and hospital pressures, some doctors, though a wrong attitude, prescribe unnecessary antibiotics for viral infections such as the flu. However, doctors can avoid such a negative attitude and simultaneously satisfy their patients by giving appropriate time to patients’ complaints, taking a medical history, and conducting a detailed clinical examination [37]. In this study, about half of the students showed a positive attitude towards not expecting antibiotics prescription from the doctor if suffering from the flu. The students’ responses improved in phase-II after attending the basic medical science courses. Another study from the general public of Saudi Arabia indicates a negative attitude towards the same [38]. This suggests that education improves the attitude towards medicine usage. 

Completing antibiotics’ full course is advised for proper recovery from bacterial infection [39]. The students around the world indicate a diverse attitude towards this issue [17,25,29]. In this study, more than 74% of students in both phases showed a positive attitude towards the statement stopping antibiotic course when feeling better. The medications retain their quality and effectiveness if used within the due expiry date; therefore, it is essential to follow the expiry date printed on the labels [40]. In contrast to the Jeddah study, most of the PPS had a positive attitude towards checking the expiry date before use; nevertheless, their attitude did not improve significantly in phase-II [3]. We could not find any notable reason for the difference in attitudes between Riyadh and Jeddah PPS. Keeping stocks of antibiotics at home for future use and using leftover antibiotics are negative attitudes, and also examples of self-medication [41]. In this study, a substantial number of students had a positive attitude about keeping antibiotics stock at home and about using leftover antibiotics for respiratory illness. Our findings match Italy’s study; on the other hand, 78.9% of Jeddah students showed a negative response towards keeping leftover antibiotics [3,20].

## 5. Conclusions

This article describes the effects of basic medical science courses on PPSs’ knowledge and attitude about antibiotics. PPSs’ knowledge and attitude about antibiotics generally improved in phase-II but at a significant level only with the statements regarding the level of knowledge, paracetamol considered as an antibiotic, and antibiotic resistance phenomenon due to over-usage. The level of knowledge about antibiotics amongst PPS is comparatively higher than other studies conducted in Saudi Arabia and nearly identical to medical students’ knowledge from Italy. The PPS curriculum requires review to obtain further improvement in students’ knowledge. Furthermore, the PPS could be approached for prescribing antibiotics during or after graduation; therefore, courses in their curriculum should focus on different antimicrobial therapy aspects.

The limitation of this study is that it was a cross-sectional study; it could have been a more critical study if conducted on a cohort.

## Figures and Tables

**Table 1:** Summary of demographic characteristics.

Characteristics	Course	Number	Percentage (%)
Age			
19.87 ± 1.67	Pre	421	97.0
20.15 ± 1.85	Post	396	93.6
Gender			
Male	Pre	233	53.8
	Post	234	55.3
Female	Pre	200	46.2
	Post	189	44.7
Highest educational status of the father			
Primary or lower	Pre	32	7.4
	Post	27	6.4
Secondary	Pre	111	25.6
	Post	83	19.8
College/University	Pre	291	67.0
	Post	310	73.8
Highest educational status of the mother			
Primary or lower	Pre	57	13.1
	Post	52	12.4
Secondary	Pre	113	26.0
	Post	107	25.5
College/University	Pre	264	60.9
	Post	261	62.1
Monthly income			
<5000 SAR	Pre	24	5.6
	Post	20	5.1
5000–15,000 SAR	Pre	109	25.5
	Post	87	22.1
>15,000 SAR	Pre	290	67.9
	Post	286	72.8

**Table 2:** Usage and source of antibiotics.

Course	Recent Use (within 6 Months)	Number	Percentage (%)
Pre	Yes	176	40.6
	No	258	59.4
Post	Yes	147	34.8
	No	275	65.2
Source of antibiotic			
Pre	Prescribed	134	78.8
	Without prescription	36	21.2
Post	Prescribed	117	80.7
	Without prescription	28	19.3

**Table 3:** Level of knowledge.

Level of Knowledge	Total Score	n (%)		McNemar-Bowker Test
		Pre	Post	
Poor	0–4	25 (5.8%)	27 (6.4%)	0.044
Moderate	5–9	229 (53.4%)	182 (43.4%)	
Good	10–14	175 (40.8%)	210 (50.1%)	

**Table 4:** Association of demographic characteristics with level of knowledge.

Characteristics	Course	Level of Knowledge			*p* Value
		Poor (0–4)	Moderate (5–9)	Good (10–14)	
Gender					
Male	Pre	12 (5.2%)	123 (53.0%)	97 (41.8%)	0.287
	Post	19 (8.7%)	104 (47.5%)	96 (43.8%)	
Female	Pre	13 (6.6%)	105 (53.6%)	78 (39.8%)	
	Post	8 (4.0%)	77 (38.7%)	114 (57.3%)	
Educational status of father					
Primary or lower	Pre	6 (18.7%)	15 (46.9%)	11 (34.4%)	0.742
	Post	3 (9.4%)	17 (53.1%)	12 (37.5%)	
Secondary	Pre	4 (3.6%)	64 (58.2%)	42 (38.2%)	
	Post	8 (7.5%)	43 (40.2%)	56 (52.3%)	
College/University	Pre	15 (5.2%)	150 (52.3%)	122 (42.5%)	
	Post	16 (5.7%)	122 (43.6%)	142 (50.7%)	
Educational status of mother					
Primary or lower	Pre	8 (14.3%)	26 (46.4%)	22 (39.3%)	0.392
	Post	1 (1.8%)	24 (42.1%)	32 (56.1%)	
Secondary	Pre	8 (7.1%)	53 (47.3%)	51 (45.5%)	
	Post	9 (8.1%)	49 (44.1%)	53 (47.7%)	
College/University	Pre	9 (3.4%)	150 (57.5%)	102 (39.1%)	
	Post	17 (6.8%)	109 (43.4%)	125 (49.8%)	
Monthly income					
<5000 SAR	Pre	4 (16.7%)	13 (54.2%)	7 (29.2%)	0.604
	Post	3 (11.5%)	9 (34.6%)	14 (53.8%)	
5000–15,000 SAR	Pre	7 (6.5%)	59 (55.1%)	41 (38.3%)	
	Post	5 (4.6%)	55 (50.9%)	48 (44.4%)	
>15,000 SAR	Pre	14 (4.9%)	152 (52.8%)	122 (42.4%)	
	Post	19 (6.8%)	115 (41.4%)	144 (51.8%)	

The level of statistical significance was set at *p* < 0.05.

**Table 5:** Change in knowledge statements.

Statement	Course	Correct Answer	Incorrect Answer	Unsure	*p* Value (McNemar-Bowker Test)
Role of Antibiotic					
Antibiotics are medicines that can kill bacteria.	Pre	390 (91.3%)	16 (3.8%)	21 (4.9%)	1.000
	Post	380 (90.9%)	20 (4.8%)	18 (4.3%)	
Antibiotics can be used to treat viral infections.	Pre	298 (69.5%)	81 (18.9%)	50 (11.6%)	0.356
	Post	305 (72.8%)	87 (20.8%)	27 (6.4%)	
Antibiotics can cure all infections.	Pre	365 (85.1%)	15 (3.5%)	49 (11.4%)	0.303
	Post	341 (82.2%)	29 (7.0%)	45 (10.8%)	
Antibiotics are indicated to relieve pain/inflammation.	Pre	122 (28.8%)	224 (52.8%)	78 (18.4%)	0.202
	Post	131 (31.6%)	221 (53.3%)	63 (15.2%)	
Antibiotics are used to stop fever.	Pre	180 (42.8%)	129 (30.6%)	112 (26.6%)	1.000
	Post	193 (46.8%)	137 (33.3%)	82 (19.9%)	
Identification of Antibiotic					
Penicillin is an antibiotic.	Pre	325 (76.3%)	43 (10.1%)	58 (13.6%)	0.133
	Post	331 (80.5%)	41 (10.0%)	39 (9.5%)	
Aspirin is a new generation of antibiotic.	Pre	175 (41.7%)	62 (14.8%)	183 (43.5%)	0.072
	Post	201 (48.9%)	80 (19.5%)	130 (31.6%)	
Paracetamol is considered as an antibiotic.	Pre	179 (42.4%)	106 (25.1%)	137 (32.5%)	<0.001
	Post	235 (56.5%)	91 (21.9%)	90 (21.6%)	
Diphenhydramine is not an antibiotic.	Pre	58 (13.7%)	65 (15.4%)	299 (70.9%)	0.702
	Post	65 (15.9%)	74 (18.1%)	270 (66.0%)	
Dangers of Antibiotic					
Overuse of antibiotics can cause antibiotic resistance.	Pre	272 (65.4%)	117 (28.1%)	27 (6.5%)	0.003
	Post	313 (76.0%)	75 (18.2%)	24 (5.8%)	
Antibiotics may cause allergic reaction.	Pre	338 (80.7%)	25 (6.0%)	56 (13.3%)	0.440
	Post	315 (78.0%)	26 (6.4%)	63 (15.6%)	
All antibiotics do not cause side effects.	Pre	387 (91.5%)	8 (1.9%)	28 (6.6%)	0.071
	Post	361 (87.4%)	17 (4.1%)	35 (8.5%)	
Completion of Treatment Course					
You can stop taking a full course of antibiotic if your symptoms are improving.	Pre	320 (74.9%)	72 (16.9%)	35 (8.2%)	0.871
	Post	313 (76.0%)	67 (16.2%)	32 (7.8%)	
The effectiveness of treatment is reduced if a full course of antibiotic is not completed.	Pre	350 (82.4%)	26 (6.1%)	49 (11.5%)	0.640
	Post	346 (84.2%)	25 (6.1%)	40 (9.7%)	

The level of statistical significance was set at *p* < 0.05.

**Table 6:** Change in attitude statements.

Statement	Course	Agree	Disagree	Unsure	*p* Value (McNemar-Bowker Test)
When I get a cold, I will take antibiotics to help me get better more quickly.	Pre	68 (15.9%)	318 (74.3%)	42 (9.8%)	0.716
	Post	57 (13.7%)	317 (76.0%)	43 (10.3%)	
I expect antibiotics to be prescribed by my doctor if I suffer from common cold symptoms	Pre	113 (26.3%)	204 (47.6%)	112 (26.1%)	0.277
	Post	87 (20.9%)	213 (51.1%)	117 (28.0%)	
I normally stop taking antibiotics when I start feeling better.	Pre	84 (19.7%)	320 (74.6%)	25 (5.7%)	0.128
	Post	75 (18.1%)	313 (75.3%)	28 (6.7%)	
If my family member is sick, I usually will give my antibiotics to them.	Pre	14 (3.3%)	395 (92.7%)	17 (4.0%)	0.015
	Post	35 (8.4%)	365 (88.0%)	15 (3.6%)	
I normally keep antibiotics stocks at home in case of an emergency.	Pre	101 (23.6%)	276 (64.6%)	50 (11.8%)	0.285
	Post	84 (20.3%)	280 (67.8%)	49 (11.9%)	
I will use leftover antibiotics for a respiratory illness.	Pre	18 (4.2%)	374 (88.0%)	33 (7.8%)	0.235
	Post	31 (7.5%)	355 (86.2%)	26 (6.3%)	
I will take antibiotics according to the instruction on the label.	Pre	386 (90.6%)	23 (5.4%)	17 (4.0%)	0.044
	Post	356 (86.2%)	24 (5.8%)	33 (8.0%)	
I normally will look at the expiry date of antibiotics before taking it.	Pre	341 (80.0%)	43 (10.1%)	42 (9.9%)	0.263
	Post	338 (81.6%)	33 (8.0%)	43 (10.4%)	
When a family member or a friend feels sick, I recommend antibiotics.	Pre	27 (6.3%)	353 (82.3%)	49 (11.4%)	0.457
	Post	31 (7.5%)	325 (77.9%)	61 (14.6%)	

The level of statistical significance was set at *p* < 0.05.

## Data Availability

The data presented in this study are available upon request from the corresponding author.
